# Serological follow-up after syphilis diagnosis in Israel

**DOI:** 10.1017/S0950268824000566

**Published:** 2024-04-12

**Authors:** Galia Grauenfels Cohen, Matan J. Cohen, Ze’ev Dveyrin, Zohar Mor, Efrat Rorman, Ehud Kaliner

**Affiliations:** 1 School of Public Health, Tel Aviv University, Tel-Aviv Jaffa, Israel; 2 Faculty of Medicine, Hebrew University, Clalit Health Services, Jerusalem district, Israel; 3 National Public Health Laboratory Tel Aviv, Ministry of Health, Tel-Aviv Jaffa, Israel; 4 Central Department of Health, Ministry of Health, Ramla, Israel; 5 School of Health Sciences, Ashkelon Academic College, Ashkelon, Israel

**Keywords:** syphilis, surveillance, follow-up, continuity of care, serology

## Abstract

The global incidence of syphilis is increasing. Continuity of care challenges the control of sexually transmitted diseases. In this study, we assessed the follow-up and serological decline differences between community- and hospital-diagnosed patients in Israel. A historical cohort study was conducted using the Israel National Syphilis Center (NSC) repository. Patients with a positive non-specific Venereal Disease Research Laboratory (VDRL) test between 2011 and 2020 were included. Rates of serological follow-up and serological titre decreases were compared between hospital- and community-diagnosed patients. The study included 4,445 patients, 2,596 (58.4%) were diagnosed in community clinics and 1,849 (41.6%) in hospitals. Of community-diagnosed patients, 1,957 (75.4%) performed follow-up testing, compared with 834 (51.2%) hospital-diagnosed patients (*p* < 0.001). On multivariate analysis, the odds ratio of serology follow-up among community-diagnosed patients was 2.8 (95 per cent confidence interval (95% CI): 2.2–3.5) that of hospital-diagnosed patients. There were 1,397 (71.4%) community-diagnosed patients with serological titre decrease, compared with 626 (74.9%) hospital-diagnosed patients (*p* = 0.03). On multivariate analysis, this difference diminished. Serological follow-up testing is suboptimal and was performed more often among patients initially diagnosed in the community compared to hospitals. Continuity of care should be improved to promote successful patient care and prevent disease spread.

## Introduction

In recent years, there have been repeated reports of the resurgence of sexually transmitted infections (STIs); among them is the age-old syphilis [1]. Since the middle of the twentieth century, penicillin has been introduced to treat syphilis, and validated surveillance and follow-up tests and protocols were distributed [2–4]. Yet, challenges still exist due to multiple healthcare service providers and fragmented responsibilities of medical institutions in screening, notifying, and treating syphilis and follow-up. Additionally, the social stigma augments patients’ sense of shame and preference for obscurity, with some patients choosing to receive medical care from different providers and disrupting open communication [5].

In Israel, syphilis is a notifiable disease. Since 2010, there have been documented increases in the incidence of syphilis infections, including primary, secondary, and latent events, as well as a rise in congenital syphilis cases. The recorded incidence of primary and secondary syphilis is 3.4 cases per 100,000, and the recorded incidence of congenital syphilis is 0.04 per 100,000 [6].

The Centers for Disease Control and Prevention recommends either the traditional algorithm or the reverse algorithm for syphilis screening. The traditional algorithm starts with a nontreponemal test, and if positive (or reactive), samples are confirmed by a specific treponemal test. The reverse algorithm starts with a specific treponemal test, and if positive (or reactive), samples are confirmed by a nontreponemal test. Discordant samples are resolved with a second specific treponemal test, preferably based on different antigens than the original treponemal test [7–11].

The National Syphilis Center (NSC) at the National Public Health Laboratory uses the reverse algorithm for syphilis testing and confirmation. All specific treponemal-positive samples in Israel are sent to the NSC for confirmation and Venereal Disease Research Laboratory (VDRL) test titre examination. This regulatory process is performed routinely and provides the setting for national data analysis.

All Israeli citizens are medically insured by one of the four healthcare medical organizations (HMOs) that provide community services in Israel and pay for hospital care and outpatient clinics. When patients are transferred between providers, there is a potentially flawed flow of information and the continuity of care may be breached. As continuity of care is critical to successful treatment and follow-up, this study aimed to assess the differences in follow-up rates between patients who were diagnosed in community clinics versus those who were diagnosed in hospitals.

## Methods

This historical cohort study was based on data collected at the NSC. The study included patients with positive confirmatory non-treponemal-specific VDRL tests collected between 1 January 2010 and 31 December 2021. Only patients who were first diagnosed with specific treponemal tests between 1 January 2011 and 31 December 2020 were included. Anonymously performed tests were excluded, since follow-up in other settings cannot be fulfilled.

The institution of diagnosis was designated as either community HMO clinics, hospitals, or other medical institutions in which non-anonymized tests were performed, such as private laboratories and blood donation clinics. Additional data available included age, sex, nationality, ethnicity, and place of residence.

The primary study outcome was the percentage of serological syphilis follow-up tests of newly diagnosed VDRL-positive patients, as recorded in the national laboratory data set. This was assessed by examining the existence and timing of the first repeated test sent for confirmation, after the initial diagnostic-specific syphilis test. The secondary outcome was the percentage of significant serological decline after the first diagnostic VDRL test results among patients for whom follow-up tests were sent. A significant serological decline was defined as a follow-up test either documenting at least a fourfold decline in serological titre or seroconversion.

### Laboratory methods

Tests to diagnose syphilis were performed according to a reverse algorithm using three tests, two treponemal-specific (enzyme immunoassay (EIA) and *Treponema pallidum* (*T. pallidum*) haemagglutination assay (TPHA)) and a non-treponemal (VDRL).

EIAs: They distinguish serological, specific, and indirect enzymatic reactions for the detection of antibodies against *T. pallidum*, the causative agent of syphilis, in serum or cerebrospinal fluid (CSF) samples. The test is performed automatically by Captia™ Syphilis TA Kit of Trinity Biotech on the Aesku Systems SQ II, Dynex Technologies (DS2). The device is activated with the help of the DS Matrix software, which is adapted and programmed to perform the tests at the NSC according to the instructions detailed in the supplied kit.

TPHA: It is an indirect but specific serological test, used to detect antibodies against the causative agent of syphilis, in serum and/or CSF samples. Microsyph TPHA200 Kit of Axis Shield is used for test performance according to National Public Health Laboratory procedures.

VDRL: It is a non-specific serological test, used when working with serum and/or CSF samples, to confirm cases suspected of being actively infected with syphilis. The VDRL Antigen Kit of Arlington Scientific is used for the VDRL test.

### Statistical methods

Proportions of follow-up rates were compared by the chi-square test, and two-sided *p*-values below 0.05 were considered statistically significant. A logistic regression model was performed to assess the association between independent variables and the performance of follow-up and titre decline. The model included all collected variables and generated odds ratios (ORs) and 95 per cent confidence intervals (95% CIs).

The main analysis presented assessed follow-up at least 90 days after diagnosis, as a benchmark time. Different clinical guidelines recommend the first follow-up test be performed 30, 90, or 180 days after treatment [2–4]. An additional analysis examined whether a 30- or 180-day benchmark led to different results.

The study was approved by the Institutional Review Board of the Ministry of Health (MOH-023-2022). The requirement for consent was waived by the ethics committee due to the anonymous retrolective study design.

## Results

There were 5,262 patients who were diagnosed with syphilis and were confirmed with a positive VDRL test during the study period. After filtering out those diagnosed in 2010 and those diagnosed in 2021, a total of 4,445 patients confirmed by VDRL were included in the study. For 2,859 (64.3%) diagnosed patients, we found follow-up tests sent 90 days or more after the initial diagnosis. A breakdown of the follow-up rates according to patient characteristics is presented in Table 1.

**Table 1.** Follow-up serology per patient characteristics

		Follow-up	No follow-up	
		n = 2,859 (64.0%)	n = 1,586 (36.0%)	*p*-value
Age	Under 15	15 (0.5%)	32 (2.0%)	
	15 to 24	298 (10.4%)	124 (7.8%)	
	25 to 34	1,073 (37.5%)	348 (21.9%)	
	35 to 44	770 (26.9%)	401 (25.3%)	<0.001^a^
	45 to 54	378 (13.2%)	225 (14.2%)	
	55 to 64	171 (6%)	135 (8.5%)	
	65 and older	102 (3.6%)	138 (8.7%)	
	Missing	52 (1.8%)	183 (11.5%)	
Sex	Female	501 (17.5%)	442 (27.9%)	
	Male	2,311 (80.8%)	973 (61.3%)	<0.001^a^
	Missing	47 (1.6%)	171 (10.8%)	
Source institution	HMO	195 (17.8%)	639 (40.3%)	
	Hospital Service	834 (76%)	797 (50.3%)	<0.001
	Other Laboratories	68 (6.2%)	150 (9.5%)	
Nationality	Israeli Jew	2067 (72.3%)	446 (28.1%)	
	Israeli Arab	140 (4.9%)	54 (3.4%)	<0.001^a^
	Non–citizen	148 (5.2%)	44 (2.8%)	
	Missing	504 (17.6%)	1,042 (65.7%)	
Year of initial diagnosis	2011	161 (5.6%)	160 (10.1%)	
	2012	186 (6.5%)	160 (10.1%)	
	2013	228 (8%)	151 (9.5%)	
	2014	248 (8.7%)	152 (9.6%)	<0.001^a^
	2015	272 (9.5%)	189 (11.9%)	
	2016	362 (12.7%)	149 (9.4%)	
	2017	330 (11.5%)	176 (11.1%)	
	2018	393 (13.7%)	129 (8.1%)	
	2019	362 (12.7%)	137 (8.6%)	
	2020	317 (11.1%)	183 (11.5%)	
Titre at diagnosis	1:1	668 (23.4%)	564 (35.6%)	
	1:2	427 (14.9%)	342 (21.6%)	
	**1:4**	351 (12.3%)	185 (11.7%)	
	**1:8**	262 (9.2%)	123 (7.8%)	
	**1:16**	457 (16%)	155 (9.8%)	
	**1:32**	450 (15.7%)	133 (8.4%)	
	**1:64**	204 (7.1%)	62 (3.9%)	
	**1:128**	36 (1.3%)	17 (1.1%)	
	**1:256**	2 (0.1%)	2 (0.1%)	
	**1:512**	1 (0%)	1 (0.1%)	<0.001^a^

Abbreviation: HMO, health management organization.

a Same results including/excluding patients with missing data. HMO = health management organization.

Among patients diagnosed in the community, 1,957 (75.4%) performed follow-up serological testing, compared with 834 (51.2%) patients diagnosed in hospitals (*p* < 0.001). Follow-up rates in adolescents and young adults were higher than those in older participants, in particular when compared to individuals over the age of 55. Males were recorded with a higher rate of follow-up than females. Follow-up tests were performed more frequently among Israeli Jews compared with non-citizens or non-Jewish Israeli citizens. From 2011 to 2018, there was an upward trend in performing serological follow-up. There was also a consistent upward trend of follow-up with an increasing diagnostic titre at diagnosis.

In the multivariate analysis, patients who were diagnosed in the community or at other institutions had higher follow-up rates than those who were diagnosed in hospitals (OR = 2.78, 95% CI: 2.24–3.47) (Table 2). When the analysis was limited to patients whose initial titre results were 1:8 or higher, the OR was 3.58 (95% CI: 2.61–4.9). Age over 35 was associated with reduced follow-up rates, as were female sex and Israeli citizens who were non-Jewish. The inclusion of socioeconomic ranking did not affect model results (data not shown).

**Table 2.** Regression analysis for documented follow-up

	OR	95% CI	*p*-value
Year^a^	0.7	0.69 to 0.77	<0.001
age			
Under 15	1.9	0.23 to 16.0	0.54
15 to 24	0.9	0.64 to 1.34	0.67
25 to 34	1	REF	
35 to 44	0.7	0.51 to 0.88	0.004
45 to 54	0.4	0.30 to 0.57	<0.001
55 to 64	0.3	0.23 to 0.50	<0.001
65 and older	0.2	0.10 to 0.23	<0.001
Nationality			
Israeli Jew	1	REF	
Israeli non–Jew	0.5	0.37 to 0.76	<0.001
Non–citizen	0.7	0.47 to 1.05	0.088
Sex			
Female	1	REF	
Male	2.0	1.49 to 2.63	<0.001
Original institution			
HMO	2.78	2.24 to 3.47	<0.001
Hospital	1	REF	
Other	0.8	0.45 to 1.41	0.442
1:1 and 1:2	1	REF	
1:4	1.7	1.18 to 2.41	<0.001
1:8	1.6	1.06 to 2.28	0.024
1:6	1.4	1.03 to 1.88	0.033
1:32	1.7	1.23 to 2.36	<0.001
1:64 and higher	2.1	1.44 to 3.18	<0.001

Abbreviations: CI, confidence interval; HMO, health management organization; OR, odds ratio.

a Estimate of the average change in calendar year (same from 2013 to 2014 or from 2014 to 2015).

Approximately 18% of first follow-up tests were sent between 30 and 60 days after the diagnostic test, 30% up to 90 days, and 57% up to 180 days from the diagnosis. At one year after diagnosis, a 74% follow-up rate was recorded.

**Figure 1.**
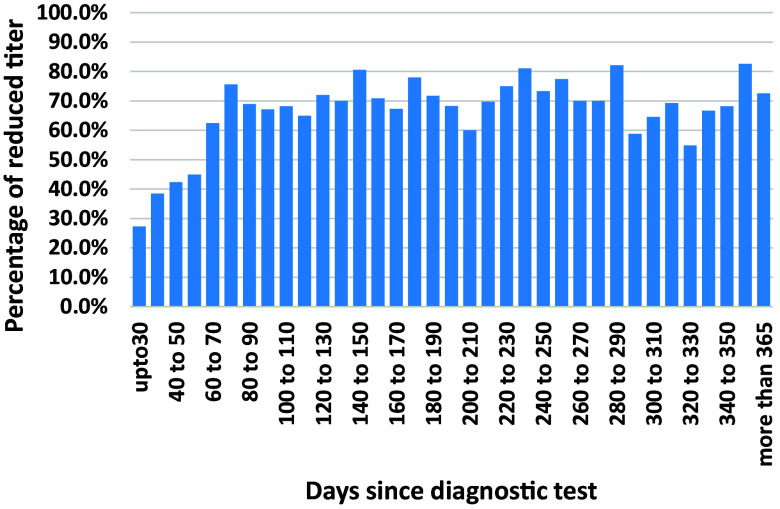
Percentage of significantly reduced serological titre of the first follow-up test, per the timing of days since the diagnostic test.

Of all the first follow-up tests sent 90 days or later after the date of diagnosis, a significant serological decline was recorded in 72.2% of the patients. With a 30-day benchmark, this rate was 65.5%, and with a 180-day benchmark, the rate was 76.2%. At 30 and 90 days since diagnosis, the rates of decrease in the serological follow-up were slightly higher (*p* < 0.05) in cases where the diagnosis was made in a hospital (Table 3). At 180 days since diagnosis, the difference between hospital and community was negligible. Tests sent up to 90 days after diagnosis had lower rates of serological decrease (Figure 1). Beyond 90 days, there was no clear trend.

**Table 3.** Rates of reduced titre (>4) per study cohort and laboratory of diagnosis

Study cohort	Laboratory	Reduced titre	No reduced titre	*p*-value
30 days	HMO	1,301 (64.2%)	727 (35.8%)	
	Hospital	633 (71.0%)	259 (29.0%)	<0.001
	Other	33 (39.8%)	50 (60.2%)	
90 days	HMO	1,397 (71.4%)	559 (28.6%)	
	Hospital	626 (74.9%)	210 (25.1%)	0.03
	Other	42 (61.8%)	26 (38.2%)	
180 days	HMO	1,396 (75.7%)	447 (24.3%)	
	Hospital service	603 (77.9%)	171 (22.1%)	0.09
	Other	39 (66.1%)	20 (33.9%)	

Abbreviation: HMO, health management organization.

In the multivariate analysis, females and non-citizens showed a less significant reduction in the serological titres, as did patients with an initial titre of 8 or higher. No association was found between the institution in which syphilis was diagnosed (HMO, hospital, other) and VDRL titre decrease (Table 4).

**Table 4.** Univariate and multivariate analysis for reduced titre^a^ on follow-up

	Univariate analysis			Regression model		
	Reduced titre	No reduced titre	*p*-value	OR	95% CI	*p*-value
Age						
Under 15	14 (0.7%)	1 (0.1%)		1.9	0.24 to 15.94	0.54
15 to 24	228 (11.2%)	71 (9.2%)		1	0.71 to 1.42	0.99
25 to 34	815 (40%)	257 (33.4%)	<0.001	1	REF	
35 to 44	539 (26.5%)	230 (29.9%)		0.8	0.65 to 1.07	0.15
45 to 54	258 (12.7%)	120 (15.6%)		0.7	0.49 to 0.89	0.007
55 to 64	121 (5.9%)	50 (6.5%)		0.8	0.51 to 1.16	0.21
65 and older	62 (3%)	41 (5.3%)		0.5	0.30 to 0.90	0.02
Nationality						
Israeli Jew	1,584 (89%)	484 (84%)		1	REF	
Israeli non–Jew	111 (6.2%)	29 (5.0%)	<0.001	1.2	0.75 to 1.85	0.47
Non–citizen	85 (4.8%)	63 (10.9%)		0.6	0.38 to 0.79	0.001
Sex						
Female	270 (13.2%)	232 (30.2%)	<0.001	0.5	0.34 to 0.59	<0.001
Male	1773 (86.8%)	537 (69.8%)		1	REF	
Original institution						
HMO	1,397 (67.7%)	558 (70.4%)		0.9	0.70 to 1.13	0.34
Hospital	626 (30.2%)	209 (26.4%)	0.024	1	REF	
Other	42 (2.0%)	26 (3.3%)		0.8	0.39 to 1.54	0.47
Original titre						
Below 8	830 (40%)	616 (77%)	<0.001	1	REF	
8 or higher	1,235 (60%)	177 (23%)		4.6	3.68 to 5.69	<0.001

a A significant serological decline was defined as a follow-up test either documenting at least a fourfold decline in serological titre or seroconversion.

## Discussion

The follow-up rates of patients who were diagnosed with syphilis in the community were higher than those who were diagnosed with syphilis in hospitals or other institutions. Differences found in the serological decrease between patients who were diagnosed in the community versus those who were diagnosed in other institutions were negligible.

The results demonstrate that continuity of care was diminished when patients were diagnosed in hospitals, as expected. There is a concern that lack of serology follow-up is associated with the possibility of loss to follow-up and, perhaps, suggests limited compliance with treatment. Professional guidelines from different parts of the world [2–4] suggest several time intervals for serological follow-up after a syphilis diagnosis and treatment. Therefore, 30- and 180-day benchmarks were also examined. These repeated analyses had no profound effect on the results, validating our conclusions regarding reduced follow-up rates in hospital-diagnosed patients.

Continuity of care and reliable flow of patient information is a recognized challenge in the care of patients treated at several institutions, as is often the case among patients diagnosed in hospitals with conditions that require ambulatory care follow-up. Diagnosis in hospital settings occurs in acute medical conditions and as part of clinical workup and, paradoxically, is associated with lower follow-up completion.

The rates of reduced titre, reaching 76% at 6 months, are also troubling as they represent the data set of the entire Israeli population and not a select high-risk group. Compared to high-risk groups and study populations, our findings are reasonable, though there is certainly a need for improvement. In a systematic review of 20 studies [12], titre decline was found to range between 20% and 99% (most commonly above 80%) with follow-up times between 6 and 24 months. This review covered a variety of studies, conducted in North America, Europe, China, Taiwan, and sub-Saharan Africa, most of which had a high prevalence of human immunodeficiency virus (HIV) infections among their study participants. A better appreciation of our results requires a more in-depth review of patient records to appreciate the degree to which they represent a lack of treatment, treatment failure, and other clinical circumstances.

We report national follow-up rates and titre reduction rates. We found no comparable syphilis follow-up surveillance data from countries similar to Israel in their characteristics and found limited data that relate to World Health Organization (WHO) programmes to reduce/eliminate congenital syphilis [13]. The Israeli NSC uses the TPHA test in its routine assessment of all sent specimens. Though the *T. pallidum* particle agglutination (TPPA) test has been shown to be superior in all disease stages [11, 14], TPHA is an accepted procedural test [1, 3, 15, 16] and serves worldwide in the diagnosis and screening of syphilis. Reduced sensitivity compared to the TPPA suggests that on a national level, there were missed opportunities for correct diagnosis and treatment.

Following the STI and HIV 2023 World Congress, the WHO released a new strategic paper that includes recommendations aiming to end acquired immunodeficiency virus (AIDS), viral hepatitis, and STIs by 2030. The strategic plans include recommendations for centralized information systems to enhance and improve the surveillance and case management of STIs. The cooperation and data sharing between stakeholders and institutions are therefore encouraged [17].

This study is subject to several limitations. First, examining the results of the study with a dichotomous outcome overestimates proactive and conscious serological surveillance rates. It is reasonable to assume that some of the repeated tests recorded in this study as follow-up tests were sent as diagnostic tests to identify new events of infection and not necessarily tests that had a rationale behind them seeking to confirm a response to treatment. Second, the available data do not include access to the patient’s medical files; therefore, it was not possible to abstract information regarding decision-making processes and the medical treatment provided. Furthermore, HIV status or other conditions bearing on clinical care and follow-up were not available in our assessment. Third, a decrease in serological titre is a recognized phenomenon also in the absence of treatment of syphilis and challenges the follow-up of syphilis patients. The stage of syphilis disease and organ involvement have a bearing on the rate of natural decline in VDRL titre, as does the initial titre value. Additionally, response to therapy will manifest in different serological declines as per disease stage and initial titre. Last, the serofast phenomenon is recognized among some patients appropriately treated, whose serology titre does not decrease after treatment [18, 19]. This phenomenon is non-differential and therefore conservative. Considering these factors, we feel that the reported declines in titre should be considered carefully as to their generalizability and internal validity.

## Conclusions

This report illuminated gaps in the continuity of care of patients diagnosed with syphilis. The lower follow-up rates among patients who were diagnosed in hospitals highlight the need for the implementation of programmes aimed at optimizing patient care. The research findings point to the need for renewed systemic thinking and initiative to improve dealing with the rising rates of syphilis in Israel.

## Data Availability

The data repository supporting this study is not available publicly due to legal limitations and is under the care of the Israeli Ministry of Health.
